# Natural Language Generation and Understanding of Big Code for AI-Assisted Programming: A Review

**DOI:** 10.3390/e25060888

**Published:** 2023-06-01

**Authors:** Man-Fai Wong, Shangxin Guo, Ching-Nam Hang, Siu-Wai Ho, Chee-Wei Tan

**Affiliations:** 1 Department of Computer Science, City University of Hong Kong, Hong Kong, China; mfwong29-c@my.cityu.edu.hk (M.-F.W.); cnhang3-c@my.cityu.edu.hk (C.-N.H.); 2 Shenzhen Research Institute, City University of Hong Kong, Shenzhen 518057, China; sxguo2-c@my.cityu.edu.hk; 3 Teletraffic Research Centre, University of Adelaide, Adelaide, SA 5005, Australia; siuwai.ho@adelaide.edu.au; 4 School of Computer Science and Engineering, Nanyang Technological University, Singapore 639798, Singapore

**Keywords:** software naturalness, large language models, AI-assisted programming

## Abstract

This paper provides a comprehensive review of the literature concerning the utilization of Natural Language Processing (NLP) techniques, with a particular focus on transformer-based large language models (LLMs) trained using Big Code, within the domain of AI-assisted programming tasks. LLMs, augmented with software naturalness, have played a crucial role in facilitating AI-assisted programming applications, including code generation, code completion, code translation, code refinement, code summarization, defect detection, and clone detection. Notable examples of such applications include the GitHub Copilot powered by OpenAI’s Codex and DeepMind AlphaCode. This paper presents an overview of the major LLMs and their applications in downstream tasks related to AI-assisted programming. Furthermore, it explores the challenges and opportunities associated with incorporating NLP techniques with software naturalness in these applications, with a discussion on extending AI-assisted programming capabilities to Apple’s Xcode for mobile software development. This paper also presents the challenges of and opportunities for incorporating NLP techniques with software naturalness, empowering developers with advanced coding assistance and streamlining the software development process.

## 1. Introduction

The advent of Big Code has become increasingly relevant in today’s software development landscape as the size and complexity of software systems continue to grow [1]. Big Code refers to the vast collection of online software artifacts such as source code repositories, bug databases, and code snippets. It represents a wealth of knowledge and experience that researchers can draw upon to improve the quality and efficiency of their own projects. The goal of Big Code is to build tools and techniques that can assist software engineers to analyze, understand, and make predictions about large codebases in a scalable and efficient manner. Big Code also has the potential to revolutionize artificial intelligence (AI) development by unitizing Big Code data. The development of statistical programming systems involves the utilization of advanced programming languages, powerful machine learning techniques such as large language models (LLMs), and natural language processing (NLP) techniques based on the software naturalness hypothesis [2]. This hypothesis posits that computer programs written in diverse programming languages can be comprehended and manipulated similarly to NLP’s treatment of human natural languages.

By employing this combination of tools, probabilistic models of extensive codebases can be constructed. These systems query a probabilistic model and calculate the most probable predictions to solve a specific challenge [3], which are then presented to the developer. In other words, the programming language is regarded as the natural language for the NLP techniques in this study. There are several crucial areas of fundamental research focused on advancing probabilistic models of “Big Code” using statistical and machine learning methodologies. By considering source code as a series of tokens and leveraging the inherent patterns and structures within vast code repositories, NLP techniques can be developed to enhance AI-assisted programming tasks, including code generation, code completion, code refinement, code summarization, defect detection, and clone detection.

AI-assisted programming can enable software engineers to work more efficiently and effectively [4], especially in situations where complex algorithms are being used that involve large amounts of code (i.e., Big Code regime). It also strikes a balance between productivity and ensuring safety, security, and reliability within the programming development environment [5]. In fact, this can even lead to the development of AI-based predictive analysis that allows human developers to more easily interact with code using natural language commands and queries as part of the software development process [6]. AI-based predictive analysis [7] can also more accurately anticipate potential issues throughout the software development life cycle and flag critical incidents [8] before they occur [9,10].

Several recent reviews have explored specific topics related to LLMs, such as fairness and bias [11], interpretability [12], explainability [13], and privacy preservation [14]. However, this review focuses primarily on language models with software naturalness. In Table 1, a detailed comparison of other reviews that have examined related topics is provided. This review also delves into the analysis of the publicly available Big Code dataset, which is designed to assist programming with AI. This review addresses the process of using language models for assessing software naturalness and examines the concept of evaluating language models using entropy. Additionally, the latest developments in AI-assisted programming using transformer-based LLMs trained on Big Code are explored, and both the generation and comprehension aspects are discussed. The review concludes with the open challenges and opportunities in AI-assisted programming. This review paper highlights the unique contributions of this review in comparison to existing reviews.

Reviews have emphasized the significance of AI-assisted programming, leading to significant advancements in this critical field of study. However, the essential components of AI-assisted programming have been presented separately, resulting in a fragmented understanding of the topic. Despite this, these independent studies have created an opportunity to view AI-assisted programming from a more comprehensive perspective. In light of this, our survey aims to provide a more structured approach to framing AI-assisted programming that extends beyond the examination of individual research topics. By doing so, this review paper hopes to offer a more comprehensive understanding of this field, highlighting the interdependencies between different areas of research.

The remainder of this review article is structured as follows. Section 2 provides an overview of the background knowledge in Big Code and software naturalness, covering topics such as the available dataset, tokenization process, existing language models, and the measurement of language models using entropy. Section 3 explores recent applications of LLMs trained with Big Code in AI-assisted programming tasks. Section 4 discusses the potential challenges and opportunities associated with LLMs in this context. Finally, Section 5 concludes the study and outlines possible directions for future work in this field.

## 2. Background

### 2.1. Main Big Code Dataset

Researchers have successively released a large amount of Big Code to train LLMs. Most datasets used to train LLMs can be applied into different tasks such as code generation and code summarization. LLMs use unsupervised learning and require large amounts of high-quality and diverse data to achieve high accuracy and generalization in their predictions. Access to large-scale, high-quality, diverse, and representative datasets is essential for developing high-performing LLMs on software naturalness. The datasets found in the literature are described in Table 2, which were accessed on 18 May 2023.

### 2.2. Tokenization

**Figure 1:**
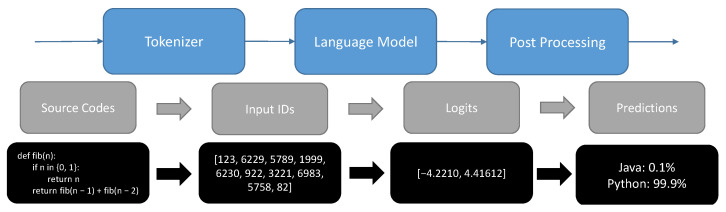
Pipeline of language models on software naturalness.

Figure 1 illustrates the pipeline of language models on software naturalness. Similar to other neural networks and raw text, language models cannot process source code directly, so the first step of the standard pipeline is to convert the code inputs into numbers of which the model can make sense. To do this, a tokenizer can be used to split the input into code syntax keyword, variables, or symbols (similar to punctuation) that are called tokens. Each token is mapped to an integer in the next step. These tokens typically correspond to words, punctuation marks, or other meaningful elements of the text. Tokenization is an important step in many NLP tasks, as it allows machine learning algorithms to process and analyze text in a more efficient and meaningful way. Some popular tokenizers are available to be used directly such as Byte-Pair Encoding (BPE) [43] and RoBERTa [44].

In the tokenization process, each token is assigned a unique identifier or index which can be used to represent the token in a numerical format that can be understood by machine learning models. Different tokenization strategies may be used depending on the specific task at hand, such as splitting text into words, phrases, or even individual characters. One common challenge in tokenization is dealing with ambiguity or variability in the text. For example, words may have different meanings depending on the context in which they appear, or may be misspelled or abbreviated in unpredictable ways. There are various techniques that can be used to address these challenges, such as using contextual information or statistical models to help disambiguate the text.

### 2.3. Language Models on Software Naturalness

**Figure 2:**
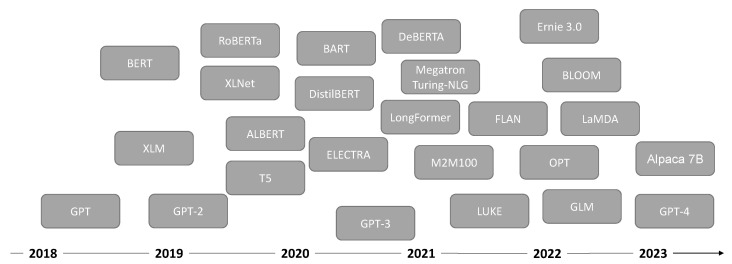
Timeline for the development of transformer-based large language models.

In this section, some of the leading transformer-based language models are presented. Figure 2 displays the timeline of the evolution of LLMs since 2018.

**Figure 3:**
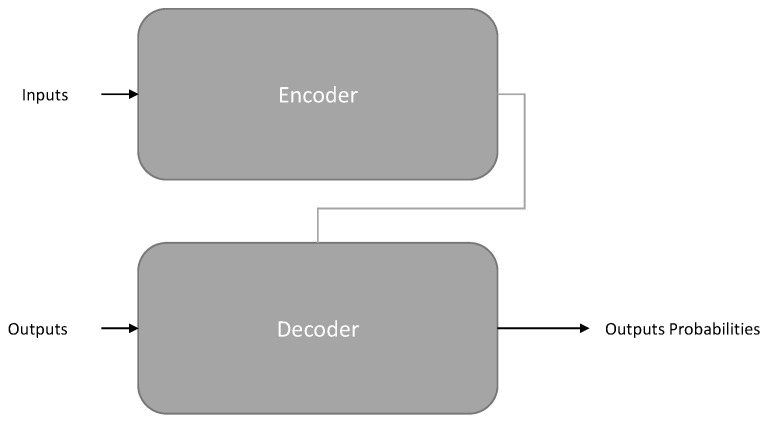
Encoder–decoder architecture. The model is primarily composed of two blocks: The encoder receives an input and builds a representation of its features, while the decoder uses the encoder’s representation along with other inputs to generate a target sequence.

**Figure 4:**
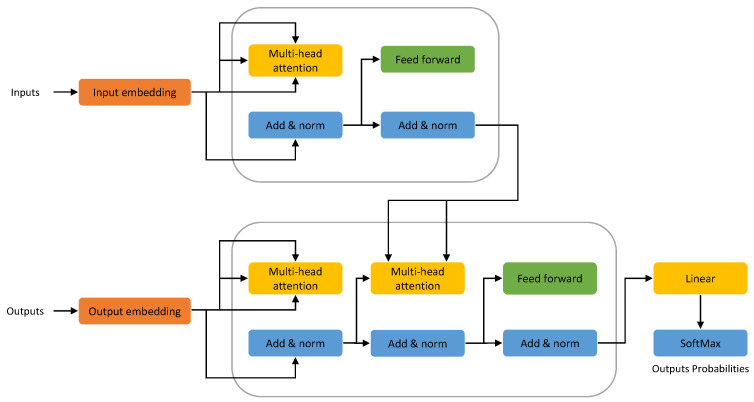
Transformer architecture. The transformer architecture retains a similar structure to that of the encoder–decoder architecture. The encoder considers all words in a sentence, while the decoder works sequentially. Once the initial words are predicted, they are used to generate subsequent words. The attention layers in the encoder consider all the words in a sentence, while the decoder works sequentially and can only focus on the words it has already translated.

Table 3 provides a summary of transformer-based language models used in AI-assisted programming. Transformer-based models are a type of neural network architecture used in NLP and other machine learning tasks. The transformer maintains a similar architecture as the encoder–decoder architecture shown in Figure 3, but the models use a self-attention mechanism to weigh the importance of different parts of the input sequence, allowing them to capture dependencies between all parts of the sequence, as shown in Figure 4. They can be parallelized more easily than previous models, resulting in faster training and lower inference times. The transformer model is one of the most well-known transformer-based models and has been used in various NLP tasks. Recently, large transformer-based models such as GPT-4 [45] and LLaMA [46] have achieved state-of-the-art performance in many benchmarks. The transformer’s ability to capture long-range dependencies is heavily reliant on dot-product attention with softmax normalization, leading to a quadratic space and time complexity in relation to sequence length, which can be a hindrance for longer inputs. This study focuses on transformer-based models for AI-assisted programming tasks.

Encoder–decoder models [47] refer to sequence-to-sequence models, utilizing both components of the transformer architecture [48]. The encoder’s attention layers can access all words in the input sentence at each stage, while the decoder’s attention layers can only access the words preceding a given word in the input. Sequence-to-sequence models such as BART [49], T5 (Text-to-Text Transfer Transformer) [50], and TreeGen [51] are well-suited for tasks that involve generating new text based on an input, such as code generation, code refinement, defect detection, and clone detection, for AI-assisted programming tasks.

Encoder-only models, also known as autoencoders, use only an encoder network to transform input data into a compressed representation. They are commonly used in unsupervised learning tasks such as dimensionality reduction and anomaly detection in NLP tasks. In the past, code embedding approaches could be utilized to obtain the representation from the input data such as Neural Network Language Model [52], Code2Vec [53], ELMo [54], TextRank [55], and GGNN [56]. For AI-assisted programming tasks, they are used for understanding tasks to learn useful representations with the BERT [57] and RoBERTa [44] of data in an unsupervised manner, which can be used as features for downstream tasks such as code translation and code summarization.

Decoder-only models, also known as autoregressive models, are a type of neural network architecture used in natural language processing tasks such as GPT-2 [58], GPT-3 [59], GPT-J [60], Reformer [61], and GPT-Neo [62], which use the decoder to predict the next token output given all previous tokens. They rely solely on a decoder network to generate output text, predicting the probability distribution of the next token given the previously generated tokens. Although they are simpler and more efficient than encoder–decoder models, they may not be as effective in tasks requiring a deeper understanding of the input–output sequence relationship. Nevertheless, they are still widely used in various natural language processing tasks for AI-assisted programming, such as code generation and code completion, and have demonstrated impressive performance in several benchmarks.

### 2.4. Measurement of Language Models with Entropy

Language models on software naturalness are trained on large code corpora and used to predict the next token in the code given its context. Mathematically, assuming a set of program tokens T and a set of program sequences S, the set of possible systems is S⊂S. A language model is a probability distribution p(.) over systems s∈S:

(1) $$\begin{array}{c} \forall s\in S\left[0<p\left(s\right)<1\right]\wedge \sum_{s\in S}p\left(s\right)=1. \end{array}$$

An estimated language model known as a pre-trained language model [63] is created by computing a maximum-likelihood estimation (MLE) of the parameter of a suitably chosen parametric distribution p(·) given a corpus *C* of programs C⊆S. This process is described in Section 2.2. The tokenization of the code is defined by the programming language to estimate the probability distribution of code tokens given the preceding context. It uses this information to make predictions or decisions in the software engineering tasks. The models are trained to predict the probability distribution of words in a sequence, based on the previous words in that sequence [64]. The language model is typically constructed using *N*-gram models, which have a long history in statistical language modeling and are widely used for estimating the probability distribution of words or characters in a text sequence [65,66]. This was the standard method before the development of word vectors and distributed representations of language using Recurrent Neural Networks (RNN) [67]. Given a system *s* with a sequence of tokens $\left\{W_{1},W_{2},\ldots W_{n}\right\}$, *N*-gram models can estimate the likelihood of tokens following other tokens. As a result, the model can estimate the probability of *s* by multiplying a series of conditional probabilities:

(2) $$\begin{array}{c} p\left(s\right)=p\left(W_{1}\right)p\left(W_{2}|a_{1}\right)p\left(W_{3}|W_{1}W_{2}\right)\ldots p\left(W_{n}|W_{1}\ldots W_{n-1}\right). \end{array}$$

An *N*-gram model captures the co-occurrence patterns of words or characters in the text. Mathematically, an *N*-gram model can be represented as a set of *N*-grams, each represented as a tuple of *n* items and their associated probabilities. The probability of an *N*-gram can be estimated by the MLE based on the frequency of occurrence of the *N*-gram in a given training corpus. This also assumes a Markov property, i.e., token occurrences are influenced only by a limited prefix length of *n*. Thus, for example, in a 3-gram (n=3) model:

(3) $$\begin{array}{c} p\left(W_{i}|W_{1}\ldots W_{i-1}\right)≅p\left(W_{i}|W_{i-2}W_{i-1}\right). \end{array}$$

The probability of a word $W_{i}$ given its preceding word $W_{i-1}$ can be estimated:

(4) $$\begin{array}{c} p\left(W_{i}|W_{i-1}\right)=count\left(W_{i-1},W_{i}\right)/count\left(W_{i-1}\right), \end{array}$$

where $count(W_{i-1},W_{i})$ is the number of times the 3-gram $\left(W_{i-1},W_{i}\right)$ appears in the training corpus, and $count(W_{i-1})$ is the number of times the word $W_{i-1}$ appears in the training corpus. The models have achieved great success in recent years and have been a driving force behind recent advancements in NLP. The performance of the technique depends on the quality of the language model and the ability of the model to accurately reflect the patterns and structures of the target data. Therefore, much research effort has been devoted to improving the quality of language models for these tasks, including developing better training algorithms, larger training corpora, and better evaluation metrics.

A representative corpus of repetitive and highly predictable programs is utilized to capture regularities within the corpus in order to evaluate the naturalness of software language models. By estimating the language model from this representative corpus, it can predict the contents of new programs with high confidence, thereby minimizing the surprise associated with the new program. In NLP, this idea is often measured using perplexity or cross-entropy (log-transformed version). Given a program $p=\{w_{1},w_{2},\ldots ,w_{n}\}$, of length *n*, and a language model Θ, it assumes that the probability of the programs estimated by the model is $p_{\Theta }$, and, thus, the cross-entropy $H_{\Theta }\left(p\right)$ can be measured:

(5) $$\begin{array}{c} H_{\Theta }\left(p\right)=-\frac{1}{n}\log p_{\Theta }\left(w_{1},w_{2},\ldots ,w_{n}\right) \end{array}$$

and a formulation can be derived from Equation (2):

(6) $$\begin{array}{c} H_{\Theta }\left(p\right)=-\frac{1}{n}\sum_{i=1}^{n}\log p_{\Theta }\left(w_{i}|w_{1},w_{2},\ldots ,w_{i-1}\right). \end{array}$$

The entropy rate of a language model is utilized to assess the naturalness of the generated text [68]. It can be computed by taking the negative logarithm of the probability of each generated token. An effective model should have low entropy for the majority of programs, assigning higher probabilities (i.e., values closer to 1) to most words in the program, thereby resulting in lower absolute log values. In practice, this involves using techniques such as maximum likelihood estimation or neural networks to estimate the parameters. The final model can then be used to make predictions by calculating the probability of a given sequence of words. Estimating entropy from empirical data has been an interesting area in information theory for AI-assisted programming [69]. For example, a method for estimating entropy with a confidence interval was proposed in [70]. Another method for estimating the entropy and redundancy of a language was provided in [68]. A model weighting principle based on the minimum description length principle was applied in [71] to develop a direct estimator of the entropy rate. The estimator can be used to estimate a Bayesian confidence interval for the entropy rate using Monte Carlo techniques. Techniques for estimating the entropy rate have been reviewed in [72]. Analytical results of estimators for entropy and mutual information can be found in [73].

## 3. AI-Assisted Programming Tasks

There are two main categories of AI-assisted programming tasks related to software naturalness: generation and understanding. The former includes code generation, code completion, code translation, code refinement, and code summarization. The latter is concerned with understanding code and includes defect detection and clone detection. Researchers have made significant efforts to enhance the quality of language models for these tasks by improving pre-training schemes, increasing the size of training corpora, developing better fine-tuning datasets, and using improved evaluation metrics. The frameworks and tools developed for these specific tasks are discussed in this section, and a summary of all the frameworks reviewed is presented in Table 4.

### 3.1. Code Generation

Program synthesis, also known as source code generation, is the process of automatically generating source code from a programming language based on user-specified constraints [74,75]. This study focuses on text-to-code generation for code generation, while code-to-code generation is referred to as code translation, which is discussed in Section 3.3. The history of code generation dates back to the use of theorem provers to construct a proof of user-provided specifications and extract corresponding logical programs [76,77]. With the increasing popularity of deep learning methods, neural methods, including Long Short–Term Memory (LSTM) [78] and Recursive–Reverse–Recursive Neural Network [79], have been adopted to generate output programs with specific inductive biases given sufficient program samples. More recently, transformer-based LLMs such as GPT-3 [59] and T5 [50] have shown impressive performance in code generation tasks by leveraging contextual representations learned from large amounts of code, as well as public code sources and natural language data, to improve program synthesis. These approaches incorporate systematic pre-training and fine-tuning tasks to develop a deep understanding of code structure and meaning, making them well-suited for software development tasks. To evaluate the models for code generation tasks, different metrics are available such as pass@k [35], which measures the percentage of problems solved using *k* generated programs per problem, BLEU-4 [80], and exact match accuracy on program synthesis benchmarks such as APPS [36], MBPP [81], and CodeBLEU [50], which consider both syntactic and semantic matches based on code structure in addition to *N*-gram matches.

### 3.2. Code Completion

Code completion, also known as autocompletion, is a software development feature that suggests possible code completions as a programmer types [82]. Its goal is to save time and reduce errors by providing suggestions for method names, variable names, and even entire code snippets [83]. Previous research on code completion started with statistical language models [84,85]. Later, LSTM-based deep learning approaches were applied to the task, aiming to learn the semantic information of source code without considering its syntactic structure [86]. To address the limitations of LSTM-based language models, transformer architecture was introduced for code completion. Normally, the language models for code completion are trained using a causal language model that predicts the unknown token after a sequence of known tokens. Recent work on code completion using LLMs [35,87] has shown impressive performance on benchmarks, such as CodeXGLUE [34], compared to existing statistical language models and deep learning approaches.

### 3.3. Code Translation

Code translation is the process of converting code from one programming language to another, with the goal of migrating legacy software. While theoretically possible, building a code translator is challenging due to differences in syntax and platform APIs between programming languages. Most current translation tools are rule-based, requiring handcrafted rewrite rules applied to an abstract syntax tree (AST) derived from the input source code. However, creating such tools demands significant expertise in both the source and target languages. Recent studies have explored using statistical machine translation [88,89] as well as deep learning approaches [90,91] for programming language translation. Quality evaluation for generated functions often uses the BLEU score, while the exact match is used to compare generated output with reference ground truth.

### 3.4. Code Refinement

Code refinement, which can be referred to as automated program repair (APR), is the process of automatically fixing bugs or vulnerabilities by converting a buggy function into a correct one. Deep learning models have a strong learning capability that enables them to learn various patterns for transforming buggy programs into patched ones from large code corpora. Many studies [92,93] have demonstrated the superior performance of deep learning-based techniques over traditional template-based [94,95], heuristic-based [96,97,98], and constraint-based [99,100] APR techniques. LLM is used to generate plausible patches or modifications to a given incorrect code. The model can be trained on a large corpus of correct code to learn the patterns and structures of correct code. When LLMs are given a faulty code, the model can then generate suggestions for how to correct it as one of the downstream tasks. The LLMs for code refinement can be evaluated by CodeXGLUE [34] or HumanEval [35] as the abstracted codes or the classical APR benchmarks such as Defects4J [101] and QuixBugs [102] as real-world codes, but the understanding and generation of concrete variable and function names is still mandatory and challenging [103].

### 3.5. Code Summarization

Code summarization is a technique used to generate English descriptions of code snippets at the function level, which can then be used to generate documentation. Typically, this involves taking the source code as input and producing a natural language summary as output. In AI-assisted programming tools, code summarization can be used to analyze code and identify optimization opportunities, such as using a binary Euclid algorithm instead of a traditional modular arithmetic-based algorithm, which can significantly improve software performance. In recent years, there has been promising research into the automatic generation of natural language descriptions of programs, with studies such as [104,105,106] making notable progress in this area. The rise of deep learning, coupled with the abundance of data from open-source repositories, has made automatic code summarization an area of interest for researchers. Many of the neural approaches [107,108] use a sequence-to-sequence approach to generate source code summaries, with some models converting the source code into various types of representations, such as token-based [109,110], tree-based [111,112], and graph-based [113,114], before passing it through language models.

### 3.6. Defect Detection

As software systems increase in complexity, it becomes more challenging to identify errors. Defect detection aims to enhance software reliability by predicting whether a piece of code is susceptible to bugs or not, by detecting previously unknown errors. Rule-based approaches have been defined in existing defect detection frameworks by inferring likely programming rules from various sources such as code, version histories, and comments [91,115,116]. Statistical language models based on *N*-gram language models have also been widely used in this area [117,118,119]. More recently, many deep learning-based solutions [95,120,121,122,123,124,125] have been proposed to bridge the gap by suggesting different feature sets from which the detection framework can learn, attempting to imitate how a practitioner looks for vulnerabilities. However, LLMs, such as CodeBERT [126], have recently emerged as a promising technique in this field due to their ability to understand code structure. These models can be trained on a large corpus of error-free code and used to identify patterns and structures in source code that deviate from those learned from the error-free code as a binary classification task [127,128]. To evaluate the model predictions, accuracy, precision, recall, and F1 scores can be used.

### 3.7. Clone Detection

Clone detection involves identifying identical or similar code fragments, known as clones, within or across software systems. The goal of clone detection is to measure the similarity between two code snippets and determine if they have the same functionality. Clones can be classified into four types [129,130], with types 1–3 being syntactic clones that differ in minor ways, while type 4 clones, known as semantic clones, are difficult to detect since they have different syntax but the same semantics and, thus, require manual validation. With the increasing amount of source code, large-scale and automatic clone detection has become essential. Several tools have been developed to perform clone detection [131,132,133,134,135,136], using techniques such as comparison of the AST, tokens, or source code text. Notable clone detection datasets include BigCloneBench [25], which contains Java code snippets.

**Table 4:** Summary of language models for AI-assisted programming tasks.

Framework	Year	Task(s)	Baseline(s)	Supported Language(s)	Open Sourced
Refactory [137]	2019	Defect Detection	BLEU	Java	✗
CuBERT [138]	2020	Code Refinement, Defect Detection	BERT	Python	✓
CugLM [139]	2020	Code Completion	BERT	Java, TypeScript	✓
Intellicode [140]	2020	Code Generation, Code Completion	GPT-2	Python, C#, JavaScript, and TypeScrip	✗
Great [141]	2020	Defect Detection	Vanilla Transformers	Python	✓
TreeGEN [51]	2020	Code Generation	Vanilla Transformers	Python	✓
C-BERT [127]	2020	Defect Detection	BERT	C	✗
TransCoder [142]	2020	Code Translation	Vanilla Transformers	C++, Java, and Python	✗
GraphCodeBERT [143]	2020	Code Summarization, Code Refinement	BERT	Java	✗
Codex [35]	2021	Code Generation, Code Completion, Code Summarization, Benchmark	GPT-3	JavaScript, Go, Perl, and 6 more	✗
Copilot [144]	2021	Code Generation, Code Completion	Codex	Java, PHP, Python, and 5 more	✗
CodeT5 [145]	2021	Code Summarization, Code Generation, Code Translation, Code Refinement, Defect Detection, Clone Detection	T5	Python, Java	✓
Tfix [146]	2021	Code Refinement, Defect Detection	T5	JavaScript	✓
CodeRL [147]	2021	Code Summarization, Code Generation, Code Translation, Code Refinement, Defect Detection, Clone Detection	T5	Java	✓
TreeBERT [148]	2021	Code Summarization	Vanilla Transformers	Python, Java	✓
BUGLAB [149]	2021	Code Refinement, Defect Detection	GREAT	Python	✓
TBCC [150]	2021	Clone Detection	Vanilla Transformers	C, Java	✓
APPS [36]	2021	Benchmark	N/A	Python	✓
CodeXGLUE [34]	2021	Benchmark	N/A	Python	✓
CoTexT [151]	2021	Code Summarization, Code Generation, Code Refinement, Defect detection	T5	Python, Java, Javascript, PHP, Ruby, Go	✓
SynCoBERT [152]	2021	Code Translation, Defect Detection, Clone Detection	BERT	Ruby, Javascript, Go, Python, Java, PHP	✗
TravTrans [153]	2021	Code Completion	Vanilla Transformers	Python	✗
CCAG [154]	2021	Code Completion	Vanilla Transformers	JavaScript, Python	✗
DeepDebug [155]	2021	Defect Detection	Reformer	Java	✓
Recoder [93]	2021	Defect Detection	TreeGen	Java	✓
PLBART [156]	2021	Code Summarization, Code Generation, Code Translation, Code Refinement, Clone Detection, Detect Detection	BART	Java, Python	✗
CODEGEN [157]	2022	Code Generation	GPT-NEO & GPT-J	Python	✓
GPT-2 for APR [158]	2022	Code Refinement	GPT-2	JavaScript	✓
CERT [39]	2022	Code Generation	CODEGEN	Python	✓
PyCoder [87]	2022	Code Generation	GPT-2	Python	✓
AlphaCode [38]	2022	Code Generation	GPT	Java	✗
InCoder [40]	2022	Code Generation, Code Completion, Code Summarization	GPT-3	Java, JavaScript, Python	✓
RewardRepair [159]	2022	Code Refinement, Defect Detection	T5	Java	✓
CodeParrot [37]	2022	Code Generation	GPT-2	Python	✓
AlphaRepair [160]	2022	Code Refinement, Defect Detection	CodeBERT	Java	✓
CodeReviewer [128]	2022	Code Summarization, Code Refinement, Defect Detection	CodeT5	Java	✓
TransRepair [161]	2022	Code Refinement, Defect Detection	BLEU	Java	✗
NatGen [162]	2022	Code Generation, Code Translation, Code Refinement	CodeT5	Java, Python, Go, JavaScript, Ruby, PHP	✓
DualSC [163]	2022	Code Generation, Code Summarization	T5	Shellcode	✓
VulRepair [164]	2022	Code Refinement, Defect Detection	T5	C, C++	✓
CoditT5 [165]	2022	Code Summarization, Defect Detection	CodeT5	Java, Python, Ruby, PHP, Go, JavaScript	✓
C4 [166]	2022	Clone Detection	CodeBERT	C++, C#, Java, Python	✓
SPT-Code [167]	2022	Code Summarization, Code Completion, Code Refinement, Code Translation	CodeBERT & GraphCodeBERT	Python, Java, JavaScript, PHP, Go	✓
ExploitGen [168]	2023	Code Generation	CodeBERT	Python, Assembly	✓
Santacoder [169]	2023	Code Summarization, Code Generation	GPT-2	Python, Java, and Javascript	✓
xCodeEval [42]	2023	Benchmark	N/A	Python, Java, C++, PHP, and 8 more	✓
StarCoder [170]	2023	Code Generation, Code Completion, Code Summarization	BERT & SantaCoder	HTML, Python, Java, and 83 more	✓

## 4. Challenges and Opportunities

### 4.1. Computational Expense

Training an LLM with millions of parameters can be computationally expensive. This is because training involves processing vast amounts of data in codes and optimizing the model’s parameters to generate accurate predictions [171]. Overall, computational expense can be due to lack of training data and computing resources such as memory, GPU, or even electricity. At the same time, the quality of the training data used to train a language model is also crucial, as poor quality data or bias in the data can lead to incorrect predictions. LLMs require massive computational resources to train, fine-tune, and run, which can be a hindrance for organizations with limited hardware resources [172].

To reduce the computational expense of training LLMs, researchers and developers can employ various techniques, such as training on subsets of the data [173,174], optimizing the hyperparameters [175], and leveraging transfer learning to reuse the knowledge learned from previous tasks. These techniques can help to speed up the training process and reduce the amount of required computing resources. Instead of training the LLMs continuously, some works focus on using prompt-learning [176,177] and human feedback [178,179,180,181,182] to improve performance of the LLMs. In prompt-based learning, the prompt serves as a guide or prompt to the language model, providing it with relevant context and guidance to generate an output that is appropriate for a particular task. The prompt can be a simple sentence or a full paragraph, depending on the complexity of the task and the amount of information needed to guide the LLMs. One of the main advantages of prompt-based learning is its flexibility and ease of use. It allows users to quickly fine-tune pre-trained language models for specific tasks without requiring a large amount of task-specific data. Additionally, prompt-based learning can be used in a semi-supervised or unsupervised manner, where the prompt provides a small amount of supervision to the language model, further reducing the necessary amount of task-specific data.

### 4.2. Quality Measurement

Leveraging LLMs in AI-assisted programming tasks has enormous potential to improve software development efficiency and reduce the time and effort required to write code manually. However, several challenges need to be addressed to ensure the performance and effectiveness of LLMs. One of the primary concerns is the quality of the generated code or documentation [35], which can be impacted by the accuracy and robustness of the LLMs. While automated code generation can save time, it can also lead to poor-quality code that is difficult to maintain and may contain bugs or security vulnerabilities [183]. Therefore, it is critical to ensure that the generated code meets the desired specifications and adheres to coding standards and best practices [184]. Another significant challenge is integrating the generated code into existing software systems seamlessly [185], ensuring that it can be maintained and updated easily over time.

To address these challenges and improve the reliability and quality of LLMs in AI-assisted programming tasks, researchers and developers are exploring various approaches and techniques. These include incorporating advanced machine learning and optimization algorithms [186,187] and developing new tools and frameworks for integrating generated code into existing software systems. Some researchers have attempted to use Variational Autoencoders [188] or Generative Adversarial Networks [189] to generate synthetic data that can be used for training LLMs, but they must ensure that the performance of these generative models is robust and reliable to ensure the quality of the synthetic data. Meanwhile, it is possible to adopt active learning [190] to improve the performance of LLMs while requiring fewer labeled training instances. This approach works by allowing the model to choose the data from which it learns [191], which enables it to compute the statistically optimal way to select training data while avoiding poor-quality data, such as buggy codes, that can negatively impact model performance. One of the significant benefits of incorporating active learning into the training process is that it can help reduce the time and effort required to label large amounts of data manually, making it a cost-effective solution for many applications [192]. By selecting the most informative data points for labeling, active learning can improve the accuracy and robustness of machine learning models, even when working with limited labeled data. The integration of active learning with LLMs remains an open question in this field of study. While active learning has shown promise in improving the performance of machine learning models, including LLMs, the application of this technique to LLMs has not yet been fully explored.

### 4.3. Software Security

Software security is a critical concern in the development of the use of LLMs [193]. While LLMs have shown significant promise in a wide range of code-related tasks, they also introduce unique security challenges that must be addressed to ensure safety and security. One of the primary security concerns when using LLMs is the potential for these models to introduce vulnerabilities into the code [194]. For example, poorly designed LLMs may generate code that is prone to buffer overflow or SQL injection attacks. Another critical concern is the possibility of LLMs being manipulated or exploited to generate malicious code that can be used for cyberattacks. For instance, an attacker may use a poisoned dataset to manipulate an LLM, resulting in the generation of malicious code that can be used to exploit vulnerabilities in the software system. Also, users without programming knowledge can generate programs with a Trojan horse phishing attack.

When using LLMs for AI-assisted programming tasks, it is essential to address software security to ensure that the generated codes or documents are secure and free from vulnerabilities, as well as to ensure the integrity of the training data used to train the LLMs. Code validation and testing involve thorough validation and testing of the generated code before integrating it with real-world systems to identify and fix any security issues. Data sanitization and validation ensure that the training data are free from malicious code or sources of bias.

### 4.4. Software Piracy

Software piracy refers to the unauthorized copying, distribution, or use of copyrighted software without the permission of the software’s owner [195,196,197]. This can take many forms, including making copies of software for personal or commercial use, distributing software through unauthorized channels, or using software beyond the terms of the licensing agreement. As the field of natural language generation and statistical machine learning for Big Code and AI-assisted programming continues to grow, concerns over software piracy have arisen. The use of open source code repositories for training AI models has led to lawsuits, with companies such as Microsoft and OpenAI accused of software piracy. The issue at hand is whether the use of open source code for training LLMs violates copyright laws. While the legal implications of this issue are still being debated, it is important to consider the ethical implications as well. The use of copyrighted code without permission raises questions about fairness and equity in the development of AI-assisted programming tools [198,199]. Also, the use of user data to train these models raises concerns over privacy and data protection. As the field continues to evolve, it will be important for researchers and developers to consider these issues and work towards finding solutions that balance the benefits of AI-assisted programming with the need for ethical and legal compliance. This may include clarifying rules around secondary uses of copyrighted code, as well as developing more transparent and opt-in data policies for training AI models.

To address software piracy, one approach is to ensure that the training data used for the development of these models are legally obtained and do not violate any copyrights or intellectual property rights according to the U.S. Copyright Office [200]. Organizations can also establish clear policies and guidelines for the ethical and legal use of these technologies. For instance, developers can be required to obtain permission or licenses before using proprietary code or software in their work. Machine learning algorithms can also be trained to identify and prevent the unauthorized distribution of copyrighted material and pirated code or software.

### 4.5. Integration with Existing Tools

**Figure 5:**
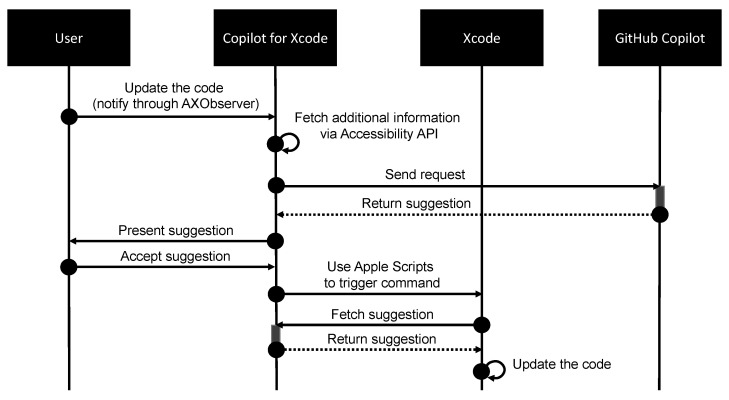
A sequence diagram of Copilot for Xcode to produce real-time suggestions with GitHub Copilot. When a user attempts to update their code, the Copilot for Xcode first receives a notification and sends a request to the GitHub Copilot API. Once the suggestions from GitHub Copilot are returned, the user can choose to adopt the suggestions and apply the changes directly to Xcode.

The opportunity to integrate tools and LLMs enhances and streamlines the software development process. By incorporating LLMs into integrated tools as cloud virtual service providers [201,202], developers can leverage the power of NLP to automate repetitive tasks, improve code quality and readability, and increase efficiency in software development. This integration can enable developers to experiment prompt engineering with public LLMs under data compliance, data security, data governance and best practices directly from their own development environment. Copilot for Xcode [203] serves as a real-world example of an application integrated with LLMs, allowing Apple developers to utilize GitHub Copilot [144] for code suggestions and ChatGPT [176] for code explanation and mutation using natural language. The connection between Xcode and Copilot is achieved by establishing communication between the Xcode source editor extension and the Copilot server, presenting suggestions in a user interface not handled by Xcode. To obtain additional information beyond the source code and file type provided by Xcode, the app utilizes the Accessibility API, which represents objects in a user interface and exposes information about each object within the application. Furthermore, for in-place code editing, the app employs the use of Apple Scripts, a scripting language in macOS for task automation, to programmatically execute extension commands and emulate menu bar interactions. The details to integrate the Copilot with Xcode are illustrated in Figure 5.

**Figure 6:**
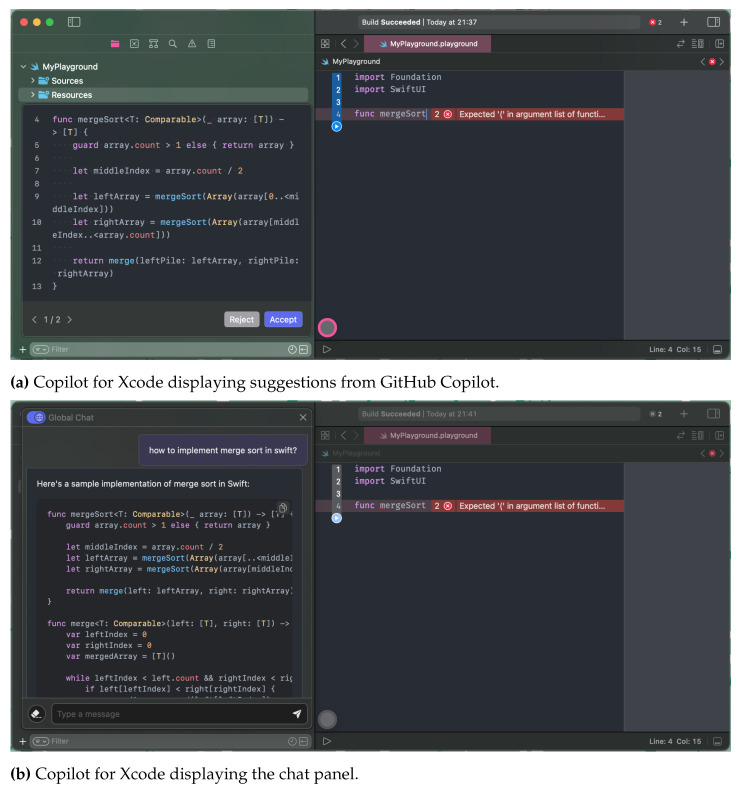
Interface of Copilot for Xcode integrated with Apple Xcode. (**a**,**b**) are the actual user interface tool, where a developer can interact with the GitHub Copilot inside the Xcode.

With these workarounds, Copilot for Xcode successfully enables Xcode to support GitHub Copilot, as shown in Figure 6. In addition, it facilitates the integration of an external chat panel that can access and read the user’s code. This chat panel serves as a connection point to leverage LLMs for functionalities such as code explanation and mutation using natural language. The chat panel can also be extended with plugins to offer additional features, including support for natural language terminal commands. The incorporation of Copilot into Xcode signifies a notable advancement in AI-powered programming for iOS/macOS, expanding the capabilities of language models to widely-used mobile software development tools.

## 5. Conclusions

This review paper explores the applications of LLMs in software naturalness to gain a better understanding of software development processes and develop applications that cater to the human aspects of software development. Firstly, it provides a background on Big Code and software naturalness, covering topics such as available datasets, tokenization processes, existing language models, and entropy-based measurements. Secondly, it summarizes recent applications of LLMs trained with Big Code in various tasks, including code generation, code completion, code translation, code refinement, code summarization, defect detection, and clone detection. Lastly, it discusses the potential challenges and opportunities associated with LLMs in the context of AI-assisted programming tasks.

Analyzing Big Code repositories and identifying patterns of naturalness can lead to more effective methods for AI-assisted programming. This can ultimately improve the quality and productivity of AI-assisted programming, making it easier for programmers to create high-quality software with fewer errors in less time. In addition to the challenges faced by LLMs for codes mentioned in this review paper, there are significant opportunities for future work in the field. These opportunities include exploring the development of LLMs that prioritize transparency and interpretability, enabling clearer explanations for code suggestions and bug fixing. Emphasizing the design of AI-assisted programming applications that prioritize fairness, transparency, and privacy is crucial, as current research tends to focus primarily on performance and efficiency. By pursuing these avenues, AI-assisted programming applications can be advanced to be more user-centric, ethically responsible, and adaptable, ultimately leading to more efficient and effective programming workflows.

## Figures and Tables

**Table 1:** Comparison of surveys on language models in software naturalness.

Title	Year	Focus Area
A Survey of Machine Learning for Big Code and Naturalness [15]	2019	Big Code and Naturalness
Software Vulnerability Detection Using Deep Neural Networks: A Survey [16]	2020	Security
A Survey on Machine Learning Techniques for Source Code Analysis [17]	2021	Code Analysis
Deep Security Analysis of Program Code: A Systematic Literature Review [18]	2022	Security
A Survey on Pretrained Language Models for Neural Code Intelligence [19]	2022	Code Summarization and Generation, and Translation
Deep Learning Meets Software Engineering: A Survey on Pre-trained Models of Source Code [20]	2022	Software Engineering
Software as Storytelling: A Systematic Literature Review [21]	2023	Storytelling
Pre-train, Prompt, and Predict: A Systematic Survey of Prompting Methods in Natural Language Processing [22]	2023	Prompt-based Learning

**Table 2:** Summary of public datasets used on Big Code. All URLs were accessed on 18 May 2023.

Dataset Name	Year	Sample Size	Language(s)	Supported Task(s)	Online URL
GitHub Java Corpus [23]	2013	14.7K	Java	Code Completion	https://groups.inf.ed.ac.uk/cup/javaGithub/
Description2Code [24]	2016	7.6K	Java, C#	Code Generation, Code Summarization	https://github.com/ethancaballero/description2code
BigCloneBench [25]	2015	5.5K	Java	Defect Detection, Clone Detection	https://github.com/clonebench/BigCloneBench
CodRep [26]	2018	58K	Java	Code Refinement, Defect Detection	https://github.com/ASSERT-KTH/CodRep-competition
CONCODE [27]	2018	104K	Java	Code Generation	https://github.com/sriniiyer/concode
WikiSQL [28]	2018	87K	SQL	Code Summarization	https://github.com/salesforce/WikiSQL
Bugs2Fix [29]	2019	122K	Java	Defect Detection, Code Refinement	https://sites.google.com/view/learning-fixes
Devign [30]	2019	26.4K	C	Code Generation, Defect Detection	https://sites.google.com/view/devign
CodeSearchNet [31]	2019	2M	Python, Javascript, Ruby, Go, Java, PHP	Code Generation, Code Summarization, Code Translation	https://github.com/github/CodeSearchNet
The Pile [32]	2020	211M	Python	Coder Generation	https://pile.eleuther.ai
CodeNet [33]	2021	13M	C++, C, Python, Java	Code Generation, Code Refinement	https://github.com/IBM/Project_CodeNet
CodeXGLUE [34]	2021	176K	Python, Java, PHP, JavaScript, Ruby, Go	Code Generation, Code Completion, Code Summarization, Defect Detection	https://github.com/microsoft/CodeXGLUE
HumanEval [35]	2021	164	Python	Code Generation	https://github.com/openai/human-eval
APPS [36]	2021	10K	Python	Code Generation	https://github.com/hendrycks/apps
Codeparrot [37]	2022	22M	Python	Code Generation	https://hf.co/datasets/transformersbook/codeparrot
CodeContests [38]	2022	13.6K	C++, Java, JavaScript, C# and 8 more	Code Generation	https://github.com/deepmind/code_contests
CERT [39]	2022	5.4M	Python	Code Generation	https://github.com/microsoft/PyCodeGPT
InCoder [40]	2022	670K	Python, JavaScript, HTML and 24 more	Code Generation, Code Summarization	https://github.com/dpfried/incoder
PolyCoder [41]	2022	1K	C, C++, Java, JavaScript, C#, Go and 6 more	Code Generation	https://github.com/VHellendoorn/Code-LMs
ExecEval [42]	2023	58K	Ruby, Javascript, Go, C++, C and 6 more	Code Sumarization, Code Generation, Code Translation	https://github.com/ntunlp/xCodeEval

**Table 3:** Summary of language models using transformers for AI-assisted programming.

Model	Type	AI-Assisted Programming Tasks
Encoder-only	Understanding	Code Summarization, Code Translation
Decoder-only	Generation	Code Generation, Code Completion
Encoder–decoder	Generation and Understanding	Code Generation, Code Refinement, Defect Detection, Clone Detection

## Data Availability

Data sharing not applicable.
